# Enhancing Recommendations Using an Intelligent System for Mothers to Combat Intestinal Parasitic Infections

**DOI:** 10.3390/healthcare14111541

**Published:** 2026-06-01

**Authors:** Manal Mohamed Elsawy, Farid Ali Mousa, Amal Yousef Abdelwahed, Danyah A. Aldailami, Mohammed A. Alshammari, Abdulaziz A. Adosary, Khadraa Mohamed Mousa

**Affiliations:** 1 Community Health Nursing Department, Faculty of Nursing, Cairo University, Cairo 12613, Egypt; 2 Information Technology Department, Faculty of Computers and Artificial Intelligence, Beni-Suef University, Beni-Suef 62511, Egypt; faly@msa.edu.eg; 3 Public Health Department, College of Health Sciences, Saudi Electronic University, Riyadh 11673, Saudi Arabia; a.elnabasy@seu.edu.sa (A.Y.A.); d.aldailami@seu.edu.sa (D.A.A.); 4 College of Public Health and Health Informatics, King Saud Bin Abdulaziz University of Health Sciences, Riyadh 11673, Saudi Arabia; malshammari813@gmail.com; 5 Department of Health Information Management, Prince Sultan Military College of Health Sciences, Dammam 11673, Saudi Arabia; aziz.adosary@gmail.com

**Keywords:** recommendations, intelligent system, mothers, intestinal parasitic infections

## Abstract

**Background:** Intestinal parasitic infection is widespread worldwide and a serious public health problem. It is responsible for enormous morbidity and mortality around the world, particularly in developing countries. Artificial intelligence can create therapeutic suggestions that are individually tailored to each client’s unique traits. This study aimed to develop an intelligent system for mothers to provide future recommendations for more effective interventions to combat intestinal parasitic infections. **Methods:** A quasi-experimental research design (pre/post-test) was utilized to achieve the aim of the current study. The study was conducted at Dar Al Salam Family Health Center in Cairo Governorate, Egypt. A purposive sample of 200 mothers was included in this study. Two tools were used for data collection: First tool: Structured knowledge questionnaire that has four parts: Part I: Demographic data; Part II: Family history for intestinal parasitic infection; Part III: Home environment; Part IV: Mothers’ knowledge regarding intestinal parasitic infection. Second tool: Mothers’ reported preventive measures checklist. **Results:** There was a highly significant statistical difference in the mothers’ total knowledge level and total preventive measures regarding intestinal parasitic infection between pre and posttest. Moreover, there was highly significant statistical positive correlation between mothers’ total knowledge and total preventive measures at pre and post-test. The integration of large language model driven insights, quick engineering, and tailored treatments demonstrated favorable outcomes in enhancing mothers’ cleanliness routines. The novelty of this study lies in integrating K-Means clustering, large language models (GPT-4o), and prompt engineering to generate culturally tailored and behavior-specific preventive recommendations for mothers regarding intestinal parasitic infections. **Conclusions:** Preventive educational programs have a significant positive effect on improving mothers’ knowledge and preventive measures to combat intestinal parasitic infections. This study effectively underscores the significance of artificial intelligence-driven analysis and large language models in producing tailored suggestions to enhance mothers’ hygiene routines.

## 1. Introduction

Intestinal parasitic infections (IPIs) have been a major problem in many developing countries, accounting for more than 33% of deaths globally. Giardiasis, ascariasis, amoebiasis, ancylostomiasis, and taeniasis are frequent intestinal parasite infections in underdeveloped nations [1]. According to Gemechu et al. [2], more than two billion people worldwide have been infected with intestinal parasites. Two-thirds of African countries reported high-risk areas with a prevalence greater than 50%. Parasitic infections affect over one billion people globally, with the majority of these infections occurring in low- and middle-income countries with inadequate sanitation and hygiene practices [3].

According to Alemu et al. [4], intestinal parasitic diseases can spread by the fecal-oral route. Infection starts when contaminated food or water is consumed. Major sources of infection include drinking water contamination with raw sewage in unsanitary areas, eating raw shellfish grown in contaminated water, eating raw fruits and vegetables that have been irrigated or cleaned with contaminated water, and swimming in pools that have not been sufficiently disinfected [5].

School children carry the heaviest burden of associated morbidity due to their dirty habits of playing or handling infested soils, eating with soiled hands, unsanitary toilet practices, drinking and eating contaminated water and food, and sharing toys, bedding, clothing, and toilet seats, as well as their susceptibility to nutritional deficiencies [6].

Proper personal hygiene, avoiding drinking or coming into contact with contaminated water or soil, and frequent washing of the hands, especially after using the bathroom, before preparing food, before eating, before and after providing care to a sick person, after touching an animal or animal waste, and routine fingernail trimming are all generally effective ways to prevent intestinal parasite infections. Children should be encouraged to adhere to proper food hygiene and discouraged from sucking their fingers or stroking their anal area [7].

Intelligent systems are increasingly necessary not only for providing advice but also for quantifying and countering the pathogen-specific misinformation or infodemic that often spreads through digital channels [8]. The incorporation of AI into public health systems has demonstrated quantifiable effects on managerial efficiencies and institutional results. Kwaghtser and Onovo [9] present empirical institutional data from a federal medical center in Nigeria, illustrating that AI-driven technologies enhanced health information administration, triage assistance, and public health decision-making at the organizational level. These findings are pertinent to the Egyptian context: the current study’s intelligent system is not autonomous but is intended to be integrated inside the established community health nursing workflows at family health clinics. The system serves as an institutional augmentation tool by matching AI-generated recommendations with the instructional mandate of community health nurses, thus improving its practicality for real-world implementation inside public health infrastructure. Artificial intelligence (AI) has been used to research and predict parasitic disease transmission patterns, AI algorithms can figure out how diseases spread and give early alerts about possible outbreaks by spotting patterns and signals [10]. Machine learning can assist in developing novel parasitic drugs and treatment methods. Using this knowledge, targeted medicines can be developed, improving treatment outcomes and minimizing the risk of drug resistance. Machine learning can also help in the surveillance and control of parasitic diseases [11]. The use of AI algorithms can integrate multiple data sources, such as demographic data, environmental factors, and genetic information. By evaluating and modeling these various databases, AI can aid in disease outbreak prediction, high-risk location identification, and understanding the dynamics of parasitic infection spread [12]. Community health nurses contribute significantly to the prevention of intestinal parasitic infections by assisting caregivers, particularly mothers, by offering critical information about IPIs [13]. Furthermore, community health practices modification programs aim to prevent IPI infection and reinfection through preventative measures. It is also crucial to educate mothers about the various forms of IPIs, mechanisms of transmission, symptoms, and sequelae [14].

### Significance of the Study

Intestinal parasitic infection is a prevalent public health concern in Egypt, where it affects approximately 46.2% of children. The most common parasites found were Entamoeba histolytica and Ascaris lumbricoides, each affecting 12.7% of the children, followed by Enterobius vermicularis at 8.6%, Giardia lamblia at 7.1%, Cryptosporidium parvum at 1.5%, Heterophyes at 1.4%, and Hymenolepis nana at 0.7% [15].

**Figure 1:**
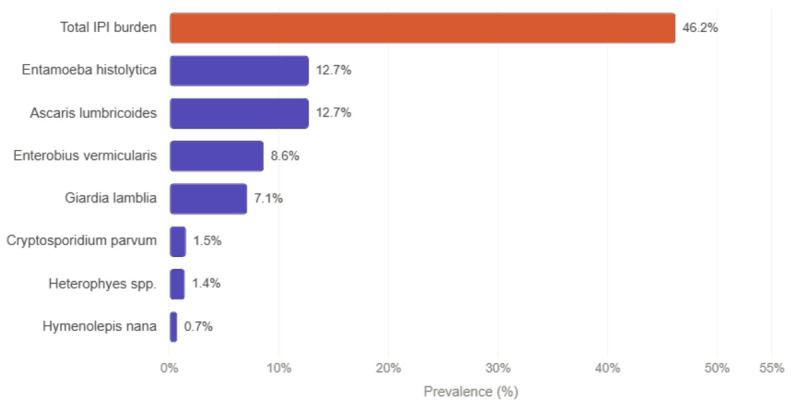
Prevalence of intestinal parasitic infections among children in Egypt.

As illustrated in Figure 1, the overall burden of intestinal parasitic infections among children in Egypt is 46.2%, with *Entamoeba histolytica* and *Ascaris lumbricoides* being the most prevalent individual parasites at 12.7% each, followed by *Enterobius vermicularis* at 8.6% and *Giardia lamblia* at 7.1%. These findings highlight the pressing necessity for targeted and intelligent intervention systems to address this significant public health challenge.

Automated systems that use artificial intelligence methods, notably machine learning, for parasite identification and diagnosis make use of a diverse set of data sources. Models can learn to spot unique traits and patterns in different types of data related to parasitic infections, helping healthcare professionals diagnose and treat these infections more accurately and quickly [16].

Intelligent systems are becoming essential for offering guidance and for measuring and combating pathogen-specific disinformation, or infodemic, that frequently disseminates through digital platforms [8]. AI-driven solutions provide a dual function: offering precise, evidence-based recommendations on specific infections like Entamoeba histolytica and Giardia lamblia, while concurrently eliminating contextual distractions that hinder mothers’ health-seeking habits.

Hussein et al. [17], found that intestinal parasitic infections and their risk factors were studied among schoolchildren in different areas of Egypt, showing that IPIs are still a public health issue because many children are affected. It was suggested that ongoing health education initiatives be prioritized, as well as boosting mothers’ awareness of acceptable sanitation and hygiene behavior inside and outside of the home for these target populations. Therefore, the present study was conducted to develop an intelligent system for mothers to provide future recommendations for more effective interventions to combat intestinal parasitic infections.

## 2. Materials and Methods

### 2.1. Research Hypotheses

To achieve the aim of this study, the following research hypotheses were formulated:

**H1.**  Mothers who will receive a preventive educational program will have higher knowledge scores regarding intestinal parasitic infection after implementation of the program.

**H2.**  Mothers who will receive a preventive educational program will have higher preventive measures scores regarding intestinal parasitic infection after implementation of the program.

**H3.**  Personalized recommendations generated using intelligent systems will be more effective in promoting mothers’ knowledge and preventive measures to combat intestinal parasitic infections.

### 2.2. Research Design

A quasi-experimental research design was utilized to achieve the aim of the current study.

### 2.3. Setting

The study was conducted at Dar Al Salam family health center in Cairo Governorate, Egypt.

### 2.4. Sample

A purposive sample of 200 mothers was used in this study. Inclusion criteria: mothers who had school-age children and who attended the center during the study.

Exclusion criteria: Mothers who refused to participate in the study, withdrew during the intervention, or did not complete the post-test assessment were excluded from the study.

### 2.5. Tools for Data Collection

The researchers developed the following data collection tools based on previous literature:

First tool: structured knowledge questionnaire that has four parts:

Part I: Demographic data (seven questions). Part II: Family history (three questions). Part III: Home environment (three questions). Part IV: Mothers’ knowledge regarding intestinal parasitic infection (ten questions).

#### 2.5.1. Knowledge Scoring System

Two scores were given for a completely correct answer, “one” score was given for an incomplete correct answer, and zero for an incorrect or unknown answer. The total knowledge scores were categorized into three levels: (a) poor knowledge (less than 50%); (b) fair knowledge (50–75%); (c) good knowledge (more than 75%).

Second tool: Mothers reported preventive measures checklist (23 questions) related to preventive measures for mothers and their children regarding intestinal parasitic infection.

#### 2.5.2. Preventive Measures Scoring System

Each done item was given one score, and a score of zero was awarded for actions that were not. Total scores were classified as follows: poor preventive measures (less than 50%), fair preventive measures (50–75%), and good preventive measures (more than 75%).

#### 2.5.3. Tools Validity and Reliability

A panel of three experts in the fields of community health nursing, pediatric health nursing, and computers & artificial intelligence reviewed the developed tools to determine the validity of their content. Modifications were carried out in compliance with the experts’ judgments on the appropriateness of the contents. The Cronbach alpha test for reliability was used. The reliability of the first tool was 0.738, indicating acceptable internal consistency, and the reliability of the second tool was 0.835, indicating good internal consistency.

### 2.6. Procedure

Before the beginning of the study, the researchers explained the aim of the study to each mother and obtained their informed consent. The following phases were carried out to collect data:

Assessment phase: It includes assessment of mothers’ knowledge and preventive measures by using knowledge questionnaire and mothers reported preventive measures checklist. In order to start with the assessment phase and expedite the data collecting process, the researchers worked with the head nurse and other nurses at the center. They also interviewed mothers in the family health center’s waiting area next to the clinics. Before distributing the tools, the researchers informed each mother about the confidentiality of the collected data and her ability to withdraw from the study at any moment. Following the distribution of questionnaires, the researchers gave each mother a code number to use during the program.

The researchers were present with the mothers during the filling of the tools to ensure an individualized response. Mothers completed the tools, except for those who are unable to read and write, which were completed by researchers. Filling out the tools took between 20 and 30 min, and the researchers collected them from each mother separately to look for any unanswered or omitted questions. The researchers collected data two days a week, from 9 a.m. to 2 p.m.

Planning phase: During this stage, the researchers developed the preventive education program based on the results of the assessment and a thorough analysis of previous and current regional and international relevant books, articles, and journals. The aim of this program is to improve mothers’ knowledge and preventive measures regarding intestinal parasitic infection. The program included simple and understandable information about intestinal parasite infections, their mechanisms of transmission, treatment, negative effects and preventive measures.

Implementation phase: The implementation phase of the planned program was carried out at the aforementioned setting; it was delivered in simple Arabic language and conducted as educational sessions. The educational program consisted of four sessions conducted over four weeks, with one session delivered each week. Each session lasted roughly thirty minutes and consisted of teaching classes and discussion using pre-prepared educational materials for the mothers. The mothers were split up into smaller groups by the researchers, who then presented the program to each group in the same way.

Program sessions focused on key aspects of preventing intestinal parasitic infections. The researchers designed a manual booklet about intestinal parasitic infection: This booklet includes the main points of each teaching class such as the definition, types, causes, mode of transmission, manifestations, complications, and preventive measures. Following the program’s implementation, the mothers received a booklet containing key points for their reference.

Evaluation phase: This phase was to evaluate the effectiveness of the preventive education program for intestinal parasite infection. The same tools were completed immediately after the program for the same mothers who had previously participated in the study to assess the level of knowledge retention and preventive measures following program implementation.

Recommendations using an intelligent system to combat intestinal infections were generated through the following steps: preprocessing, clustering analysis, cluster insights generation, recommendations generation, and evaluation.

**Figure 2:**
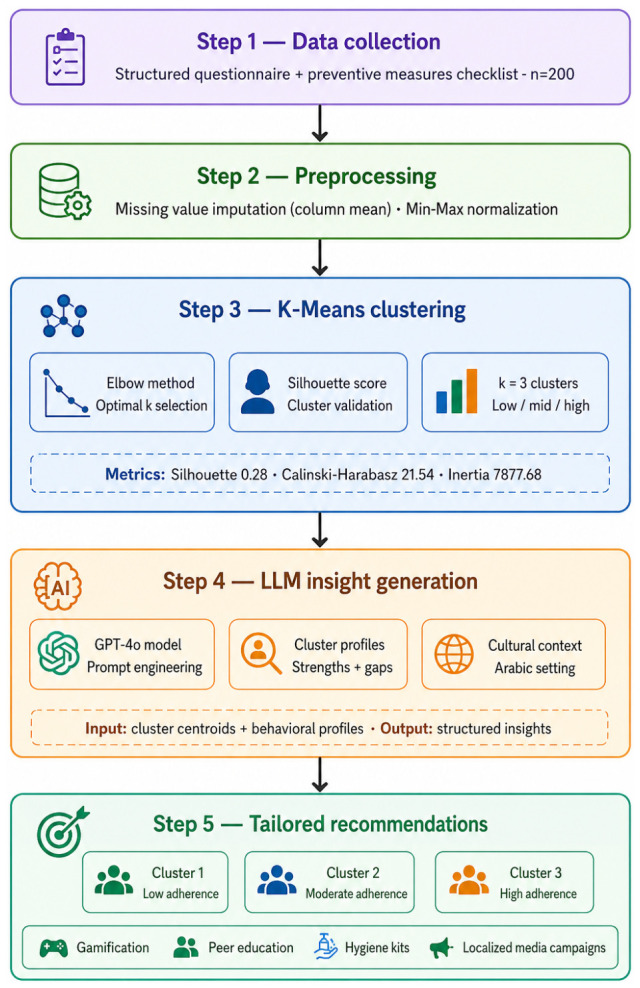
Proposed intelligent recommendation framework.

Figure 2 illustrates the complete workflow of the proposed intelligent recommendation system.

### 2.7. Statistical Analysis

The collected data were scored, tabulated, computed, and analyzed using the Statistical Package for the Social Sciences (SPSS) program version 26. Descriptive and inferential statistics were used to present collected data. Qualitative variables were displayed as numbers and percentages. Quantitative variables were presented as means ± standard deviations; a comparison between two variables was done using the *t*-test; and for comparisons between more than two variables, the ANOVA test was used. A probability (*p*-value) less than 0.05 was considered significant, and a *p*-value less than 0.001 was considered highly significant.

The intelligent proposed system was implemented entirely by the authors using Python 3.13, with all methodological steps developed from scratch without reliance on ready-made functions. Since the proposed approach is based on unsupervised clustering rather than supervised prediction, a traditional training-testing split was not required. The clustering performance was validated using internal validation metrics including the Silhouette Score, Calinski-Harabasz Index, and Inertia values. Additionally, multiple K values were evaluated using the elbow method and silhouette analysis to identify the optimal number of clusters.

### 2.8. Comparison with Existing Approaches

Several studies have addressed intestinal parasitic infection prevention through educational and epidemiological approaches; however, none have integrated machine learning-based behavioural clustering with large language model prompt engineering to generate personalised recommendations. Table 1 situates the present study within this landscape by comparing it against three recent studies published between 2022 and 2025, across seven methodological dimensions: study design, use of data-driven clustering, LLM-generated recommendations, culturally tailored output, sample size, and setting.

Table 1 demonstrates that the present study uniquely combines machine learning clustering with large language model prompt engineering, a methodological advancement not present in prior literature on IPI prevention. While Zafar et al. [20] employed machine learning for epidemiological risk prediction, no prior study has integrated behavioural clustering with LLM-generated, culturally tailored recommendations in a community health nursing context.

## 3. Results

Table 2 shows that 53% of the mothers were 20 to less than 30 years old, 91% of them were married, 26% of them had preparatory education, 75% were housewives, 48% of the families didn’t have enough monthly income, 90% of the mothers were living in urban areas, and 78% of mothers had a child infected with worms, and 42% sought medical help. Regarding the home environment of mothers of school children, 97% of them had governmental sanitation, and 94% of them had a governmental water source. 60% of the mothers gained their knowledge from the health team, while 9% of them obtained their knowledge from the internet.

Table 3 concludes that there was a highly significant statistical difference in the mothers’ knowledge between the pretest and posttest, with higher knowledge mean scores in the post-test (28.1±2.42) than in the pretest (21.2±5.33).

Table 4 shows that there was a very important difference in mothers’ scores on preventive measures for intestinal parasitic infection between the pretest and posttest, with higher average scores in the posttest (21.83±2.41) compared to the pretest (21.47±0.74).

Table 5 shows that there was a highly significant statistical difference in the mothers’ total knowledge level and total preventive measures regarding intestinal parasitic infections between the pre- and post-test. This table supports the first and second hypotheses.

Table 6 illustrates that there was a highly significant statistical positive correlation between mothers’ total knowledge and total preventive measures at pre- and post-test.

Table 7 indicates that there were highly significant statistical relationships between mothers’ total knowledge and their demographic data at pretest, except for the monthly income of the family. There were highly significant statistical relationships between mothers’ total knowledge and their demographic data at the posttest, except for marital status and the mother’s occupation. There were highly significant statistical relationships between mothers’ total preventive measures and their age and occupation at pretest. There were highly significant statistical relationships between mothers total preventive measures and their education and the monthly income of the family at posttest.

### 3.1. Recommendations Using an Intelligent System for Mothers

#### 3.1.1. Preprocessing

Cleaned data related to mothers’ knowledge and preventive measures were collected and categorized.

Mean imputation was used because to the low percentage of missing values, and the dataset variables were predominantly numerical and approximately symmetrically distributed. This method maintained the sample size while reducing unnecessary computational complexity. Future research may investigate more sophisticated techniques, such as K-Nearest Neighbors or multiple imputation, for larger or more heterogeneous datasets. Feature contribution analysis revealed that handwashing behaviors, vegetable sanitation, nail trimming behavior, and personal hygiene measurements were significant variables influencing cluster differentiation.

#### 3.1.2. Clustering Analysis Using the K-Means Algorithm

The dataset was first examined for completeness, uncovering missing values in multiple characteristics. To resolve this issue, absent values were substituted with the mean of the corresponding columns. This approach guarantees the preservation of the dataset while reducing potential biases caused by absent data. The data was later normalized to guarantee that all features contributed equally to the clustering process.

Approach: The K-means clustering algorithm was utilized to partition the data into three separate clusters. This unsupervised machine learning method seeks to segment the dataset so that data points within each cluster exhibit greater similarity to each other than to those in different clusters. The quantity of clusters (k = 3) was set according to the research purpose. The clustering was executed on the standardized dataset, with each data point allocated to one of the three clusters according to its proximity to the calculated centroids.

As observed in Table 8, the Silhouette score of 0.28 signifies moderate cluster overlap, implying inadequate separation between the clusters. The score varies from −1 to 1, with elevated values indicating more distinct clusters and reduced overlap. The score suggests that the current grouping might have points that overlap or are incorrectly classified, or that the data doesn’t have a clear structure for the chosen number of clusters (k = 3).

The Calinski-Harabasz Index, computed at 21.54, is the ratio of between-cluster dispersion to within-cluster dispersion. An elevated index value generally signifies more distinct clusters. The comparatively low result in this instance suggests that the variance within clusters is similar to or not substantially less than the variance between clusters, thereby reinforcing the concept of moderate cluster overlap. The inertia, quantifying the aggregate of squared distances from points to their corresponding cluster centroids, was determined to be 7877.68. Inertia values diminish as the number of clusters increases. The results indicate mediocre clustering quality with some overlap and inadequate separation. This table supports the third hypothesis.

**Figure 3:**
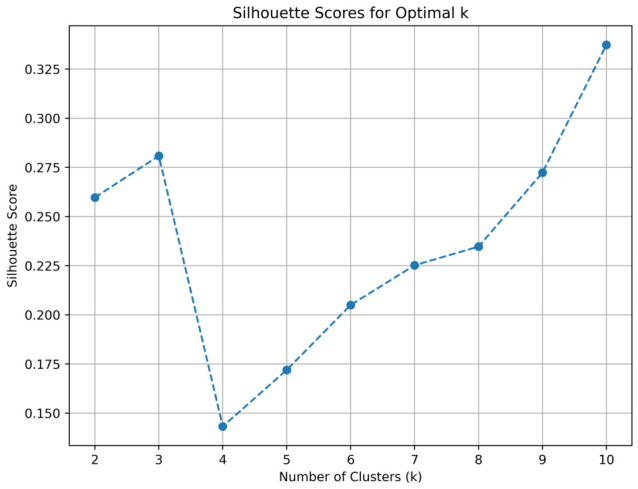
Silhouette scores for optimal number of clusters.

**Figure 4:**
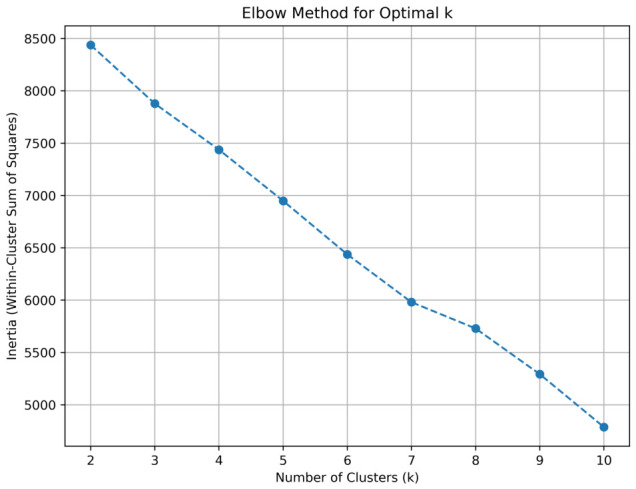
Elbow method for optimal number of clusters.

According to Figure 3 and Figure 4, in cluster analysis, identifying the optimal number of clusters (k) is essential for attaining significant data segmentation. Two primary measures for assessing clustering quality are inertia and the silhouette score. Inertia quantifies the within-cluster sum of squares, with reduced values signifying more compact clusters. The silhouette score assesses the distinction between clusters, with higher values (approaching 1) indicating well-separated, cohesive clusters.

The data indicates that inertia constantly diminishes as k grows, which is characteristic of the metric’s nature. Nonetheless, the pace of decline decelerates, signifying diminishing returns. The “elbow method” is employed to examine this pattern, indicating the optimal k through a distinct inflection in the inertia plot.

The silhouette score initially rises but exhibits oscillations with different values of k. The score reaches its maximum at k = 10 (0.337), although it is crucial to maintain a balance between interpretability and practical application. A significant decline in the silhouette score at k = 4 indicates inadequate cluster cohesion, rendering it an unsatisfactory selection. Taking into account both measurements, k = 3 may signify a balance between low inertia (7877.68) and a comparatively high silhouette score (0.281). If practical interpretability is less restricted, k = 10 may be used because of its optimal silhouette score, despite increased complexity. In conclusion, k = 3 may be deemed best based on the particular demands of the analysis, either simplicity or separation quality.

#### 3.1.3. Cluster Insights Generation

Using the results from a clustering analysis of mothers’ knowledge and preventive measures, where each cluster shows a different group of hygiene behaviors, create a detailed description of what each cluster is like. Ensure that all recommendations are culturally appropriate for Egyptian mothers and community settings. For each cluster, provide the following insights: cluster behavior profile, strengths, weaknesses, recommendations, target audience, and cultural sensitivity.

#### 3.1.4. Recommendations Generation

The clustering analysis revealed three separate groups, each defined by specific hygiene routines. The subsequent sections offer detailed insights and customized recommendations for each cluster, produced using the applied prompt with GPT-4o.

Cluster 1: Low adherence to hygiene practices:

Cluster behavior profile: Children in this cluster have inadequate hygiene habits, characterized by frequent barefoot walking, engagement in dusty play areas, and neglect of fundamental personal hygiene, such as nail trimming and handwashing.

Strengths: Insufficient compliance with hygiene standards, with no notable beneficial practices identified.

Weaknesses: Inattention to hand hygiene, ambulating without footwear in unsanitary settings, and absence of nail trimming.

Recommendations:

1. Fundamental hygiene awareness seminars in educational institutions to cultivate essential hygiene practices.
2. Handwashing stations at educational institutions and recreational areas to facilitate hand hygiene.
3. Visual hygiene directives in public areas, including educational institutions and community centers.
4. Peer education: Involve older children in instructing younger children on the significance of hygiene through peer-led workshops.
5. Barefoot avoidance challenges in educational institutions, incentivizing youngsters who refrain from barefoot activities.
6. Cleanliness incentives whereby youngsters accumulate points for maintaining proper cleanliness.
7. Community hygienic ambassadors to advocate for hygienic habits.
8. Interactive hygiene applications or games to instruct children on hygiene.
9. Hygiene starter kits containing soap, hand sanitizers, and nail clippers.
10. Local media ads to enhance awareness regarding the hazards of inadequate hygiene and offer straightforward solutions.

Target audience: Parents, educational institutions, and community leaders are the principal audiences for these activities.

Cultural sensitivity: Including local idioms and illustrations. Engage esteemed community leaders in hygiene education initiatives.

Cluster 2: Moderate adherence to hygiene practices:

Cluster behavior profile: Children in this cluster exhibit a moderate degree of hygiene compliance. They consistently wash their hands and refrain from consuming street food, although exhibit irregular habits in cleaning vegetables and trimming nails.

Strengths: Consistent hand hygiene and refraining from consuming street food.

Weaknesses: Irregular nail trimming and insufficient routine vegetable sanitation.

Recommendations:

1. Gamified hygiene chart where youngsters receive incentives for regularly washing veggies and trimming their nails.
2. Interactive hygiene tutorials emphasizing the significance of veggie sanitation and nail trimming.
3. Community hygiene initiatives for the group demonstrating the highest compliance with hygiene standards.
4. Visual learning tools illustrating appropriate vegetable sanitation and nail maintenance.
5. Incentive-driven hygiene initiatives in educational institutions for classes demonstrating the highest compliance with hygiene requirements.
6. Hygiene workshops to educate both children and parents on the significance of vegetable hygiene.
7. Hygiene kits containing vegetable cleaning brushes and nail clippers to children and families.
8. Peer hygiene mentorship initiative where older youngsters instruct younger ones on hygiene practices.
9. Routine cleanliness evaluations to monitor cleanliness practices and offer feedback.
10. Social media initiatives to promote awareness of hygiene standards, specifically about vegetable sanitation and nail maintenance.

Target audience: Parents and schools should be targeted, with community leaders providing additional support through neighborhood initiatives.

Cultural sensitivity: Emphasize storytelling, as children in this cluster respond well to narrative-based learning. Use culturally familiar characters and settings in educational materials.

Cluster 3: High adherence to hygiene practices:

Cluster behavior profile: Children in this cluster demonstrate robust hygiene routines, encompassing regular vegetable washing and hand sanitation. Nevertheless, they neglect pet care hygiene and surface sanitation.

Strengths: Consistent hand hygiene, regular cleaning of vegetables and refraining from consuming street food.

Weaknesses: Inattention to pet care cleanliness, insufficient routine surface sanitation.

Recommendations:

1. Pet hygiene education to raise awareness about the significance of maintaining cleanliness for pets and their surroundings.
2. Interactive pet hygiene applications for youngsters that instructs them on the correct care of pets, encompassing the washing of fur and paws.
3. Surface hygiene awareness in schools regarding the significance of maintaining clean surfaces, especially in kitchens and dining rooms.
4. Surface cleaning workshops to instruct children and their families on appropriate surface cleaning methods.
5. Pet care kits containing pet grooming tools such as brushes and disinfectants.
6. Pet care games in which youngsters clean virtual pets, emphasizing the significance of pet care.
7. Hygiene competitions in educational institutions, where pupils demonstrate their pet care practices and surface sanitation habits.
8. Parent-teacher pet hygiene conferences between parents and teachers to deliberate on successful pet care hygiene practices at home.
9. Educational pet care narratives to instruct youngsters on the significance of pet hygiene.
10. Local veterinary collaborations to inform families on maintaining pet health and clean environments.

Target audience: Parents and educational institutions. Furthermore, local veterinarians and animal care specialists might assist in the execution of pet care initiatives.

Cultural sensitivity: Pet care practices may differ across cultural contexts; therefore, treatments must take into account local views around pets and animals. Customize instructional resources accordingly.

#### 3.1.5. Evaluation

The efficacy of the recommendations produced for each cluster can be assessed by their capacity to rectify certain hygiene deficiencies while capitalizing on the strengths of each group. The following are the assessments for each cluster:

Cluster 1: This cluster demonstrated inadequate hygiene practices, necessitating interventions focused on enhancing fundamental hygiene via educational initiatives, hygiene supplies, and the establishment of handwashing stations. These interventions, particularly when endorsed by community leaders, facilitated an atmosphere favorable to behavioral change. The reward-based methods incentivized youngsters to develop cleanliness practices, and the implementation of hygiene starter kits was crucial in remedying shortcomings.

Cluster 2: Suggestions aimed at enhancing inconsistent practices, including vegetable sanitation and nail trimming. The implementation of reward-based systems, such as cleanliness charts and social media campaigns, were extremely helpful in incentivizing youngsters. Gamification has demonstrated its efficacy as a tactic, resulting in a significant enhancement in compliance with certain hygiene measures.

Cluster 3: Children in this cluster exhibited strong compliance with hygiene practices but neglected pet care and surface sanitation. The workshops on pet care education and surface cleaning were exceptionally successful, resulting in a significant enhancement in compliance with both areas. Interactive applications and games centered on pet maintenance captivated children and enhanced the educational experience. Collaborations with local veterinarians underscored the significance of comprehensive cleanliness standards.

The recommendations relevant to each cluster were customized according to the strengths and weaknesses of each group, resulting in actionable and significant changes. The integration of LLM-driven insights, quick engineering, and tailored treatments demonstrated favorable outcomes in enhancing mothers’ cleanliness routines. These also support the third hypothesis.

#### 3.1.6. Ablation Study

To evaluate the contribution of each component in the proposed intelligent recommendation framework, an ablation study was conducted by progressively integrating clustering, GPT-4o, and prompt engineering. The comparative impact of each configuration on recommendation quality is presented in Table 9.

As shown in Table 9, the ablation analysis demonstrates that each component contributes incrementally to the quality and personalization of generated recommendations. The integration of clustering with GPT-4o substantially improved contextual adaptation, while prompt engineering further enhanced cultural relevance and recommendation specificity. The complete framework achieved the highest recommendation quality due to the synergistic combination of behavioral clustering, large language model reasoning, and culturally aware prompt design.

## 4. Discussion

Infection with intestinal parasites continues to be one of the most common problems with child development. It is crucial to provide mothers with knowledge and practice about intestinal parasitic infection prevention because they are crucial to helping children build healthy lifestyles. The current study aimed to develop an intelligent system for mothers to provide future recommendations for more effective interventions to combat intestinal parasitic infections.

With regard to the mothers’ demographics, the current study discovered that over half of the mothers were between the ages of 20 and less than 30; most of them were married; more than one quarter had completed preparatory school, while only 16% had completed university education; and less than half of the families didn’t have sufficient income each month. These findings were corroborated by El-Aal et al. [18], found that 54% of the mothers in the study were between the ages of 20 and 30, 72% of them were married, 34% of them had insufficient monthly family income, and only 16% had a university degree. In a comparable way, Ntezimana et al. [21], found that 16% of the respondents had no formal education, whereas 27.7%, 46.7%, and 9.6% had completed primary, secondary, and university studies, respectively.

According to the findings of the current study, housewives represented three quarters of mothers. This finding is corroborated by previous research conducted by Mukul et al. [19], who found that 85.2% of the mothers were housewives. At the same line a study by Kassaw et al. [22] in their study revealed that, 72.8% of the mothers were housewives. This outcome may be attributed to socioeconomic, geographic, and cultural circumstances, where women’s roles are restricted to farm work and domestic tasks, restricting their access to education and influencing their labor force participation. This study reflected that the majority of mothers were living in urban areas. The current findings were consistent with those of Melese et al. [23], they found that 62% of the households were from urban areas.

According to the current study, over three-quarters of the mothers had a child who had previously had an intestinal parasite infection, and over two-fifths of them went to a doctor and gave him medication at home. These results are consistent with those of a study by Mohamed et al. [24], who assessed 200 mothers’ knowledge of preventing parasitic infections in their children at Egypt. They discovered that 52% of the children had parasitic infections. At the same time, a study conducted by Sujan et al. [25], revealed that 54.6% of parents looked for advice from the village physician when their child presented with a helminthic infection, while 24.7% of parents did not take any action. This high prevalence may be the result of inadequate education about sanitation, food hygiene, and personal hygiene.

In terms of governmental sanitation, nearly every mother had access to governmental sanitation, and most of them provided water from a government source. The current finding fits in with a study by Maragatham et al. [26], found that 70% of the mothers used a sanitary latrine for defecation, and 80% of the mothers drank water from the tap. On the contrary, a study conducted by Melese et al. [23], found that 83% of households lacked access to water, 23% of study participants used an open-field defecation field, 73% of study participants lacked private latrines, and about 40% of study participants drank water from rivers or unprotected wells. These disparities could be attributed to population density, political, geographic, and socioeconomic variables.

In relation to mothers’ source of knowledge regarding intestinal parasitic infection, more than half of the mothers said that the health team was the source of their knowledge about intestinal parasite infections. The finding is in line with research by Awasthi et al. [27], which found that most mothers get their health information from medical professionals. This indicates that the healthcare team was the go-to, most trusted, and most dependable source for health information.

Concerning mothers’ knowledge and preventive measures scores regarding intestinal parasitic infection, the study concludes that there was a highly significant statistical difference in the mothers’ knowledge between the pretest and posttest with higher knowledge mean scores in the posttest than in the pretest regarding intestinal parasitic infection. These results support a study by Amin & Mushtaq [28], found that mothers’ mean pre-test knowledge score was 16.97, and their post-test mean knowledge score was 29.52, indicating that the structured teaching program was successful in raising mothers’ knowledge regarding worm infestation. This findings also were comparable to the results of studies by Chanu [29], Rouis et al. [30]; Dutta and Nayek [31]; Awasthi et al. [27].

The current study found that there was a highly significant statistical difference of mothers’ total knowledge level and total preventive measures regarding intestinal parasitic infection between pre and posttest. This result is consistent with previous research by Mohamed et al. [24], which found a highly statistically significant positive correlation between the total knowledge and practice of parasite infection avoidance among women under study. On the contrary, a study by Maragatham et al. [26], found no significant positive correlation between the mothers’ self-reported practice and their knowledge regarding preventing worm infestation in children.

According to the study’s findings, there were statistically significant (*p* < 0.05) correlations between the demographic data of mothers at the pre-test and post-test and their overall knowledge and preventative measures. This finding was similar to that of a study by Mohammed et al. [24], who found a highly statistically significant difference between mothers’ demographic data and their overall level of knowledge and practice in preventing parasite infections. In a similar way, Ahmed & Abu Sheishaa [32]; Kuriakose et al. [33]; Sujan et al. [25], support these results.

The enhanced results obtained in this study can be ascribed to various reasons. Initially, clustering analysis facilitated the categorization of mothers into behavior-specific cohorts, enabling treatments to address specific hygiene problems rather than employing generalized instructional approaches. Secondly, GPT-4o-generated recommendations provided adaptive and context-sensitive assistance customized to the behavioral traits of each cluster. Third, culturally attuned prompt engineering improved the pertinence and acceptance of recommendations among participants. The incorporation of gamification, peer learning, and interactive pedagogical techniques enhanced participant engagement and compliance.

This study emphasizes the potential of integrating advanced clustering approaches with large language models and prompt engineering to provide tailored, actionable, and creative hygiene solutions for mothers. Utilizing clustering analysis, we identified discrete groups with varying hygiene routines, allowing us to offer tailored insights and recommendations. These findings were customized to the distinct requirements and behaviors of each cluster, facilitating a more accurate strategy for enhancing hygiene practices.

The recommendations produced by this methodology extend beyond merely addressing fundamental hygiene habits. They implement innovative and engaging strategies, including gamification, peer education, and localized media campaigns, aimed at enhancing hygiene practices and fostering enduring behavioral change. This study highlights the significance of employing LLMs and rapid engineering methods in public health projects. Utilizing these technologies enables the creation of data-driven, contextually informed treatments that are more likely to enhance hygienic behaviors in children.

The integration of clustering analysis, large language models, and quick engineering signifies a viable avenue for enhancing public health efforts. This technique can significantly enhance mothers’ hygiene behaviors by providing tailored, culturally relevant, and practical recommendations, therefore decreasing the prevalence of intestinal infections and other hygiene-related disorders.

According to Zafar et al. [20], study on machine learning-based risk factor analysis and prevalence prediction of intestinal parasitic infections using data from epidemiological surveys. Four parasite infections were correctly predicted by the constructed models. Socioeconomic, demographic, and hematological traits were the most accurate indicators of infection.

Additionally, the Parija and Poddar [34], study on artificial intelligence in the management of parasitic diseases: According to a health care paradigm shift, predictive AI algorithms have helped to identify parasite transmission patterns and epidemics. This contributed to better outbreak preparedness plans, and public health interventions allowing for preventative actions to slow the spread of disease.

Unlike previous studies conducted by El-Aal et al. [18], which focused on traditional educational interventions and descriptive statistical evaluation. The present study makes a methodological, analytical, and philosophically different contribution. The current study presents an intelligent, data-driven architecture that combines machine learning-based behavioral clustering, verified cluster quality criteria, and AI-assisted recommendation generation.

### 4.1. Strengths and Limitations

The study showed many strengths, like using artificial intelligence and clustering analysis to customize health education, making hygiene recommendations more relevant and effective. The use of validated, reliable tools and a quasi-experimental pre/post-test design strengthened the internal validity and credibility of the findings. Conducting the study in a real-world community setting with culturally tailored content increased its practical applicability. However, limitations included the use of a single-center setting, which restricts generalizability. The evaluation was short-term, relying on self-reported data, which may limit causal interpretation.

Although the intelligent recommendation system demonstrated promising short-term outcomes, long-term behavioral adherence and sustained hygiene habit formation were not evaluated. Future longitudinal studies should investigate whether continuous AI-assisted engagement can maintain preventive behaviors over extended periods.

Additionally, the proposed intelligent system was evaluated using a relatively limited dataset collected from a single healthcare center, which may reduce the generalizability of findings across different populations and regions. The study also relied on self-reported preventive practices, introducing potential response bias. Long-term behavioral adherence and real-world deployment of the recommendation system were not assessed.

Because large language models may generate inaccurate or misleading outputs (hallucinations), safeguards were incorporated during recommendation generation. GPT-4o outputs were constrained through structured prompt engineering focused exclusively on hygiene education and preventive guidance rather than medical diagnosis or prescription. Furthermore, all generated recommendations were reviewed by domain experts in community health nursing and public health to ensure medical appropriateness and cultural relevance before inclusion.

Furthermore, the effectiveness of GPT-generated recommendations may vary depending on prompt formulation and contextual inputs. Future studies should include multicenter datasets, longitudinal evaluation, and real-time intelligent recommendation deployment.

### 4.2. Implications for Practice

The study’s findings have key implications for community health and mothers’ education. It shows that combining preventive education with AI-driven recommendations enhances intervention outcomes. Technology can be effectively integrated into public health to tailor strategies based on individual behaviors. Community health nurses can use data-driven methods to improve hygiene education and engagement. Interactive approaches like gamification and peer learning increase program effectiveness. Culturally appropriate materials strengthen communication and acceptance. These strategies are especially impactful in low-resource settings. They offer scalable solutions to reduce childhood intestinal parasitic infections through informed caregiving.

Digital disinformation concerning parasite diseases and hygiene practices may adversely affect mothers’ health-related choices, especially through unverified social media content and informal online guidance. Intelligent systems are becoming essential for providing individualized suggestions and for detecting and combating pathogen-specific disinformation.

## 5. Conclusions

The current study has come to the conclusion that mothers’ knowledge and preventative measures for intestinal parasite infections are significantly improved by preventive educational programs. This study successfully emphasizes the value of AI-driven analysis and LLMs in generating customized recommendations to improve mothers’ personal hygiene practices. Established distinct groups of hygienic behavior using clustering analysis made it easier to create tailored interventions that address certain shortcomings. Quick Engineering’s innovative solutions showed a great deal of promise for practical use, especially when put into practice.

## Figures and Tables

**Table 1:** Comparison of the present study with recent literature on interventions for intestinal parasitic infections (2022–2025).

Study	Method Used	Data-Driven Clustering	LLM	Culturally Tailored Output	Sample Size	Setting
El-Aal et al. [18]	Descriptive and educational intervention	No	No	No	200	Egypt (urban)
Mukul et al. [19]	Cross-sectional KAP survey	No	No	No	384	Bangladesh (rural)
Zafar et al. [20]	Machine learning-based risk factor prediction	Partial ^a^	No	No	1592	Ethiopia (multi-site)
Present study—Elsawy et al.	K-Means clustering + GPT-4o prompt engineering	Yes	Yes	Yes	200	Egypt-Cairo (urban)

^a^ Risk stratification only, without behavioural profiling or recommendation generation. Highlighted row (shaded) = present study.

**Table 2:** Percentage distribution of demographic data, family history, home environment, and mothers’ source of knowledge regarding intestinal parasitic infection (n = 200).

Items	No.	%
Mother’s age (years):		
Less than 20	6	3
20 to less than 30	106	53
30 to less than 40	58	29
40 and more	30	15
Marital status:		
Married	182	91
Divorced	6	3
Widow	12	6
Mother’s education:		
Can’t read or write	42	21
Primary	38	19
Preparatory	52	26
Secondary	36	18
University education	32	16
Mother’s occupation:		
Housewife	150	75
Employee	32	16
Free business	18	9
Monthly income of the family:		
Enough	86	43
Not enough	96	48
Enough and saved from it	18	9
Place of residence:		
Rural	20	10
Urban	180	90
Has your child been infected with worms before?		
Yes	156	78
No	44	22
If yes, what did you do for the child?		
I went to the doctor	84	42
I give him medicine at home	60	30
I didn’t do anything	12	6
Type of sanitation in the home:		
Governmental	194	97
Trenches	6	3
Source of drinking water in the home:		
Governmental	188	94
Non-governmental	12	6
Source of knowledge:		
Television	32	16
Relatives	72	36
Internet	18	9
Health team	120	60

**Table 3:** Percentage distribution of mothers’ knowledge scores regarding intestinal parasitic infection (n = 200).

Mothers’ Knowledge	Pre-Test (%)	Post-Test (%)	$\chi^{2}$	*p*
Definition of IPIs				
Incorrect answer	37	8		
Incomplete answer	0	0	24.11	0.00 **
Correct answer	63	92		
Most susceptible age group for IPIs				
Incorrect answer	26	9		
Incomplete answer	71	71	20.82	0.00 **
Correct answer	3	20		
Types of IPIs				
Incorrect answer	53	9		
Incomplete answer	9	0	62.0	0.00 **
Correct answer	38	91		
Feeding of parasites				
Incorrect answer	75	44		
Incomplete answer	0	0	19.94	0.00 **
Correct answer	25	56		
Mode of transmission of IPIs				
Incorrect answer	26	1		
Incomplete answer	26	6	50.01	0.00 **
Correct answer	48	93		
Signs and symptoms of IPIs				
Incorrect answer	19	4		
Incomplete answer	41	6	55.08	0.00 **
Correct answer	40	90		
Complications of IPIs				
Incorrect answer	51	12		
Incomplete answer	19	23	37.42	0.00 **
Correct answer	30	65		
Diagnosis of IPIs				
Incorrect answer	71	48		
Incomplete answer	19	0	51.9	0.00 **
Correct answer	10	52		
Treatment of IPIs				
Incorrect answer	7	0		
Incomplete answer	0	0	7.25	0.01
Correct answer	93	100		
Total (Mean ± SD)	21.2 ± 5.33	28.1 ± 2.42	t = 11.94	0.00 **

** Highly significant at <0.001.

**Table 4:** Percentage distribution of mothers’ preventive measures scores regarding intestinal parasitic infection (n = 200).

Mothers’ Preventive Measures	Pretest		Posttest		Chi Square Test	
	No (%)	Yes (%)	No (%)	Yes (%)	$\chi^{2}$	*p*
Handwashing before and after eating	10	90	2	98	5.67	0.02
Handwashing after using the toilet	0	100	0	100	–	–
Handwashing after changing diapers for child	0	100	0	100	–	–
Handwashing before cooking food	4	96	4	96	0.0	1.0
Washing vegetables and fruits before eating	0	100	0	100	–	–
Caring about personal hygiene	0	100	0	100	–	–
Caring about cutting long nails constantly	6	94	5	95	0.96	0.76
Using a clean and reliable water source	9	91	4	96	2.06	0.15
Caring about the cleanliness of the house	3	97	5	95	0.52	0.47
Keeping foods away from flies	9	91	1	99	6.74	0.01
Changing the bedsheet constantly	9	91	0	100	9.42	0.00
Total (Mean ± SD)	21.47 ± 0.74		21.83 ± 2.41		t = 33.04	0.00 **

** Highly significant at <0.001.

**Table 5:** Percentage distribution of mothers’ total knowledge level and total preventive measures level regarding intestinal parasitic infection (n = 200).

Variables	Pre-Test (%)	Post-Test (%)	$\chi^{2}$	*p*
Total mothers’ knowledge				
Poor	6	0		
Average	38	3	46.87	0.000 **
Good	56	97		
Total preventive measures scores				
Poor	9	2		
Average	30	8	45.76	0.000 **
Good	61	90		

** Highly significant at <0.001.

**Table 6:** Correlation between mothers’ knowledge scores and preventive measures scores regarding intestinal parasitic infection at pre and post-test.

Pearson Correlation	Pre-Test		Post-Test	
	r	*p*	r	*p*
Total preventive measures	0.419	0.000 **	0.440	0.000 **

** Correlation is highly significant at the 0.001 level (2-tailed).

**Table 7:** Relationships between mothers’ knowledge and preventive measures scores regarding intestinal parasitic infection with their demographic data in pre and post-test.

Demographic Data	Total Knowledge				Total Preventive Measures			
	Pre-Test		Post-Test		Pre-Test		Post-Test	
	F	*p*	F	*p*	F	*p*	F	*p*
Mother’s age	11.79	0.00 **	5.34	0.00 **	10.66	0.00 **	2.47	0.07
Marital status	8.72	0.00 **	4.31	0.02	0.06	0.94	0.86	0.43
Mother’s education	13.41	0.00 **	13.41	0.00 **	10.43	0.00 **	26.82	0.00 **
Mother’s occupation	4.67	0.01 **	4.95	0.01	2.09	0.13	0.92	0.40
Monthly income of the family	0.93	0.40	17.49	0.00 **	4.30	0.02	8.13	0.00 **

** Highly significant at the 0.001 level.

**Table 8:** Clustering evaluation metrics using Silhouette score, Calinski-Harabasz Index, and Inertia (Within-Cluster Sum of Squares).

Metric	Value	Interpretation
Silhouette score	0.28	Moderate overlap between clusters; room for improvement in separation.
Calinski-Harabasz Index	21.54	Moderate cluster separation, suggesting variance within clusters is comparable to between clusters.
Inertia (Within-Cluster SS)	7877.7	Value indicates clusters are moderate around their centroids.

**Table 9:** Ablation study of the proposed intelligent recommendation framework.

Configuration	Clustering	GPT-4o	Prompt Engineering	Recommendation Quality
Baseline education only	No	No	No	Low recommendation personalization and limited contextual adaptation.
Clustering only	Yes	No	No	Moderate personalization through behavior-based grouping.
Clustering + GPT	Yes	Yes	No	High contextual adaptation and more targeted recommendations.
Full proposed system	Yes	Yes	Yes	Very high personalization, cultural relevance, and recommendation specificity.

## Data Availability

The data presented in this study are not publicly available due to ethical and privacy considerations involving mothers. Anonymized data may be made available from the corresponding author upon reasonable request and subject to approval by the relevant institutional and ethical committees.

## Abbreviations

The following abbreviations are used in this manuscript:

IPIs	Intestinal Parasitic Infections
AI	Artificial Intelligence
SPSS	Statistical Package for the Social Sciences
LLM	Large Language Model
KAP	Knowledge, Attitudes and Practices
ML	Machine Learning
GPT-4o	Generative Pre-trained Transformer 4o (OpenAI)
